# Identification and Validation of Cuproptosis-Related Prognostic Signature and Associated Regulatory Axis in Uterine Corpus Endometrial Carcinoma

**DOI:** 10.3389/fgene.2022.912037

**Published:** 2022-07-22

**Authors:** Yun Chen

**Affiliations:** Department of TCM Gynecology, Hangzhou TCM Hospital Affiliated to Zhejiang Chinese Medical University, Hangzhou, China

**Keywords:** uterine corpus endometrial carcinoma, cuproptosis, prognostic signature, CDKN2A, immunotherapy

## Abstract

**Background:** Uterine corpus endometrial carcinoma (UCEC) is a common gynecological malignancy globally with high recurrence and mortality rates. Cuproptosis is a new type of programmed cell death involved in tumor cell proliferation and growth, angiogenesis, and metastasis.

**Methods:** The difference in cuproptosis-related genes (CRGs) between UCEC tissues and normal tissues deposited in The Cancer Genome Atlas database was calculated using the “limma” R package. LASSO Cox regression analysis was conducted to construct a prognostic cuproptosis–related signature. Kaplan–Meier analysis was conducted to compare the survival of UCEC patients. A ceRNA network was constructed to identify the lncRNA–miRNA–mRNA regulatory axis. Quantitative reverse transcription-polymerase chain reaction (qRT-PCR) was performed to verify CRG expression in UCEC.

**Results:** The expression of FDX1, LIAS, DLAT, and CDKN2A were upregulated, whereas the expression of LIPT1, DLD, PDHB, MTF1, and GLS were downregulated in UCEC versus normal tissues. The genetic mutation landscape of CRGs in UCEC was also summarized. Gene Ontology and Kyoto Encyclopedia of Genes and Genomes analyses revealed that these CRGs were enriched in the tricarboxylic acid (TCA) cycle, glycolysis, and HIF-1 signaling pathway. LASSO Cox regression analysis was performed and identified a cuproptosis-related prognostic signature including these three prognostic biomarkers (CDKN2A, GLS, and LIPT1). UCEC patients with high risk scores had a poor prognosis with an area under the curve of 0.782 and 0.764 on 3- and 5-year receiver operating characteristic curves. Further analysis demonstrated a significant correlation between CDKN2A and pTNM stage, tumor grade, immune cell infiltration, drug sensitivity, tumor mutational burden (TMB) score, and microsatellite instable (MSI) score. The data validation of qRT-PCR further demonstrated the upregulation of CDKN2A and the downregulation of LIPT1 and GLS in UCEC versus normal tissues. The ceRNA network also identified lncRNA XIST/miR-125a-5p/CDKN2A regulatory axis for UCEC.

**Conclusion:** The current study identified a cuproptosis-related prognostic signature including these three prognostic biomarkers (CDKN2A, GLS, and LIPT1) for UCEC. The ceRNA network also identified that lncRNA XIST/miR-125a-5p/CDKN2A regulatory axis may be involved in the progression of UCEC. Further *in vivo* and *in vitro* studies should be conducted to verify these results.

## Introduction

Uterine corpus endometrial carcinoma (UCEC), originating from the endometrium, is a common gynecological malignancy globally (Yang et al., 2020a). In 2020, there is an estimated 417,367 new cases of UCEC and an estimated 97,370 disease-related deaths worldwide (Sung et al., 2021). Although the cure rate of early UCEC can reach 95%, the prognosis of UCEC was still poor, because of its high recurrence rate and high mortality rate (Wang et al., 2020; Cui et al., 2021). Furthermore, there are few satisfactory biomarkers or approaches that could predict the prognosis of UCEC patients accurately. Despite a lot of effort having been made to explore the mechanism of the tumorigenesis and progression of UCEC, the specific mechanism was not fully elucidated. Thus, identifying the prognostic signature and associated potential mechanism of UCEC is greatly significant.

Cuproptosis is a new type of programmed cell death identified by Tsvetkov et al. (2022). Excess copper could result in mitochondrial protein aggregation and cause a distinct form of cell death (Kahlson and Dixon, 2022). Copper is fundamental material in various materials in biological processes including mitochondrial respiration, iron uptake, and antioxidant/detoxification processes (Ruiz et al., 2021). Increasing pieces of evidence demonstrated the involvement of copper in tumor cell proliferation and growth, angiogenesis, and metastasis (Oliveri, 2022). Different from apoptosis, ferroptosis, pyroptosis, and necroptosis, limited studies have been conducted on the role of cuproptosis-related genes (CRGs) in the progression and prognosis of cancers, including UCEC. Hence, the present study focus on the role of CRGs in UCEC.

Competing endogenous RNAs (ceRNAs) could regulate each other via the competition for shared miRNAs at the posttranscriptional level (Qi et al., 2015). The lncRNA–miRNA–mRNA ceRNA network has been suggested to play a vital role in many types of cancers, including LUAD (Wu et al., 2020). More and more studies suggested the importance of lncRNA–miRNA–mRNA ceRNA network in the study of the molecular mechanism of malignancies (Wang et al., 2019; Chen et al., 2021a).

The Cancer Genome Atlas (TCGA, https://cancergenome.nih.gov/) is a landmark cancer genomics program that contains a lot of clinical and bioinformatics data on cancers (Yang et al., 2013). Increasing pieces of evidence have highlighted database mining as one of the promising approaches to studying the molecular mechanism and prognostic signatures of cancer. Thus, bioinformatics analyses were applied in the current study to identify cuproptosis-related prognostic signature and associated regulatory axis in UCEC.

## Materials and Methods

### Dataset and Preprocessing

The flowchart of the current study is shown in Supplementary Figure S1. Based on a previous study, a total of 10 CRGs were obtained, including FDX1, LIAS, LIPT1, DLD, DLAT, PDHA1, PDHB, MTF1, GLS, and CDKN2A (Yang et al., 2020a). Using TCGA (https://portal.gdc.cancer.gov/) database (Tomczak et al., 2015), the gene expression profile of CRGs of UCEC was isolated and R (version 4.0.5) (Kang et al., 2021) was used to perform data preprocessing with associated packages. The RNA-seq data were then normalized to transcripts per kilobase million value.

### Mutation Landscape, Gene Ontology, and Kyoto Encyclopedia of Genes and Genomes Analysis

After obtaining the single nucleotide variation (SNV) and copy number variation (CNV) data of UCEC from the TCGA database, we then explore their mutation landscape in UCEC using R software with a “maftools” package (Mayakonda et al., 2018). Seven types of mutation were included in this analysis: Missense_Mutation, Nonsense_Mutation, Frame_Shift_Ins, Splice_Site, Frame_Shift_Del, In_Frame_Del, and In_Frame_Ins. In Gene Ontology (GO) analysis and Kyoto Encyclopedia of Genes and Genomes (KEGG) pathways analysis, the “ggplot2” package (Maag, 2018) was utilized to perform this analysis, and *p* < 0.05 was set.

### Cuproptosis-Related Prognostic Signature Analysis

Prognosis analysis, including overall survival (OS), progression-free survival (PFS), and disease-free survival (DFS), was conducted to identify potential prognostic biomarkers. Log-rank test was performed to calculate the *p*-values, hazard ratio (HR), and 95% confidence interval. Using these potential prognostic biomarkers, LASSO Cox regression analysis was performed to construct a cuproptosis-related prognostic signature. After calculating the risk score of each patient with UCEC [Risk score = $\overset{4}{\underset{i}{\sum }}Xi\times Yi$ (X: coefficients, Y: candidate gene expression)], all UCEC cases were divided into two parts (low and high risks) and the survival curve of the two parts was drawn using Kaplan–Meier method. Moreover, a time-dependent receiver operating characteristic (ROC) curve was drawn using the “timeROC” package (Bai et al., 2021) to evaluate the efficiency of the prognostic signature. Moreover, a predicted nomogram was also constructed to predict the 1, 3, and 5-year DFS rates of UCEC patients considering the clinical characteristics and prognostic signature.

### Single Gene Analysis

After obtaining the abundance of immune cells using TIMER (https://cistrome.shinyapps.io/timer/), the correlation between cuproptosis-related prognostic genes and immune cells was analyzed using the Pearson correlation test (Li et al., 2017). The IC50 of 265 small molecules and its corresponding gene expression were isolated from Genomics of Drug Sensitivity in Cancer (GDSC) (Yang et al., 2013). The correlation between cuproptosis-related prognostic gene expression and drug IC50 was analyzed using the Pearson correlation test. Moreover, correlation analysis between cuproptosis-related prognostic gene expression and TMB/MSI was performed using the Spearman method.

### Cell Culture and qRT-PCR

The cells and culture conditions were as follows: Human endometriosis cell line hEM15A (Dulbecco’s modified eagle medium (DMEM) containing 10% FBS) and UCEC cell lines KLE (MEM containing 10% FBS) and ANC3A (DMEM containing 10% FBS) in a 5% CO_2_ incubator at 37°C. The total RNA of these cells was isolated from TRIzol (Invitrogen) and RNA extraction kits. The PrimeScript RT and miRNA PrimescripTM RT kits (E047-01B; Novoprotein) were used to reverse transcribe RNA into complementary DNA (cDNA). This was followed by qRT-PCR for gene expression analysis. The mRNA expression levels were normalized against glyceraldehyde 3-phosphate dehydrogenase expression.

### lncRNA–miRNA–mRNA ceRNA Network Analysis

The miRNA target of CDKN2A was explored with StarBase (http://starbase.sysu.edu.cn/) (Li et al., 2014), TargetScan (https://www.targetscan.org/) (Lin et al., 2021), and miRWalk (http://mirwalk.umm.uni-heidelberg.de/) (Sticht et al., 2018). Furthermore, the lncRNA target interacting with the miRNA target was explored with lncBase (http://carolina.imis.athena-innovation.gr/) (Karagkouni et al., 2020) and StarBase (http://starbase.sysu.edu.cn/) (Li et al., 2014). Furthermore, Student *t*-test and KM test were performed to evaluate the expression and prognosis of miRNA and lncRNA in UCEC.

## Results

### Expression of Cuproptosis-Related Genes in Uterine Corpus Endometrial Carcinoma

**FIGURE 1:**
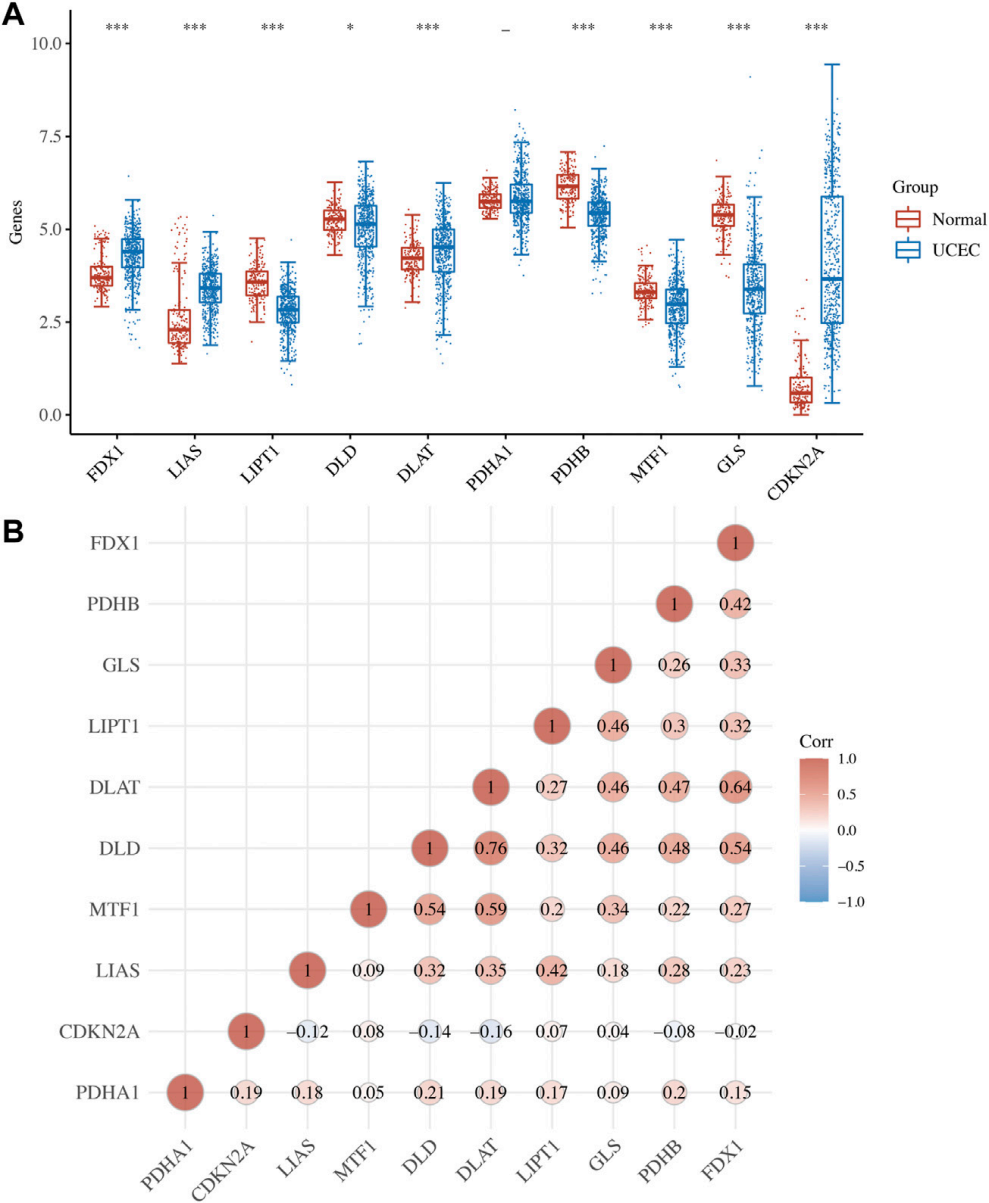
Expression of cuproptosis-related genes (CRGs) in uterine corpus endometrial carcinoma (UCEC). **(A)** The mRNA level of CRG in UCEC versus normal tissues. **(B)** The correlation between each member of CRG in UCEC. *p < 0.05; **p < 0.01; and ***p < 0.001;—*p* > 0.05.

Analysis expression revealed that 9 of 10 CRGs were differentially expressed in UCEC (Figure 1A). In a more specific sense, the expression of FDX1, LIAS, DLAT, and CDKN2A was upregulated, whereas the expression of LIPT1, DLD, PDHB, MTF1, and GLS were downregulated in UCEC versus normal tissues (Figure 1A, all *p* < 0.05). Further correlation analysis revealed that most of these CRGs were positively correlated with each other (Figure 1B).

### Mutation Landscape and Functional Enrichment Analysis

**FIGURE 2:**
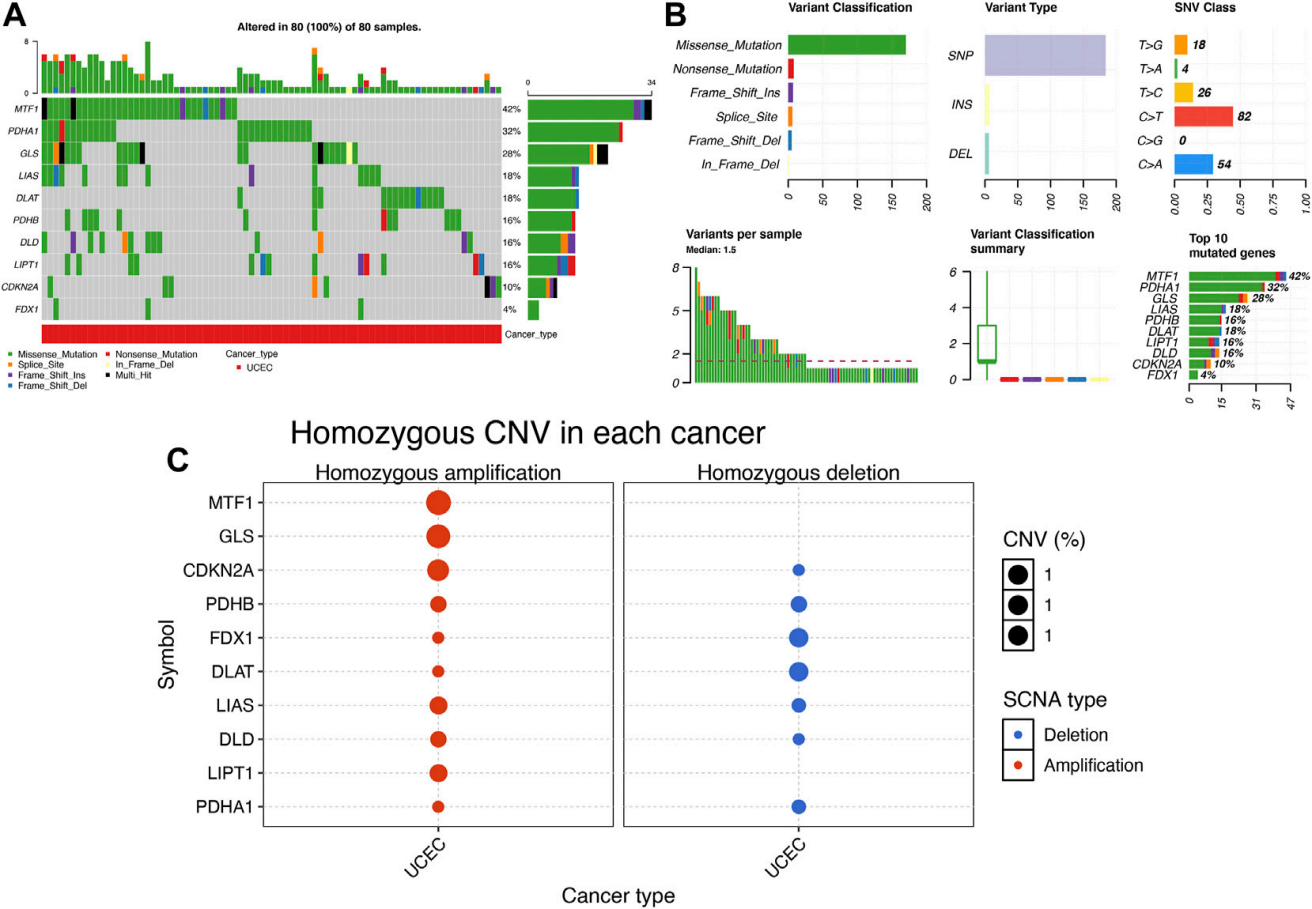
Mutation landscape of cuproptosis-related genes (CRGs) in uterine corpus endometrial carcinoma (UCEC). **(A,B)** Single nucleotide variation analysis of CRG in UCEC. **(C)** Copy number variation analysis of CRG in UCEC.

**FIGURE 3:**
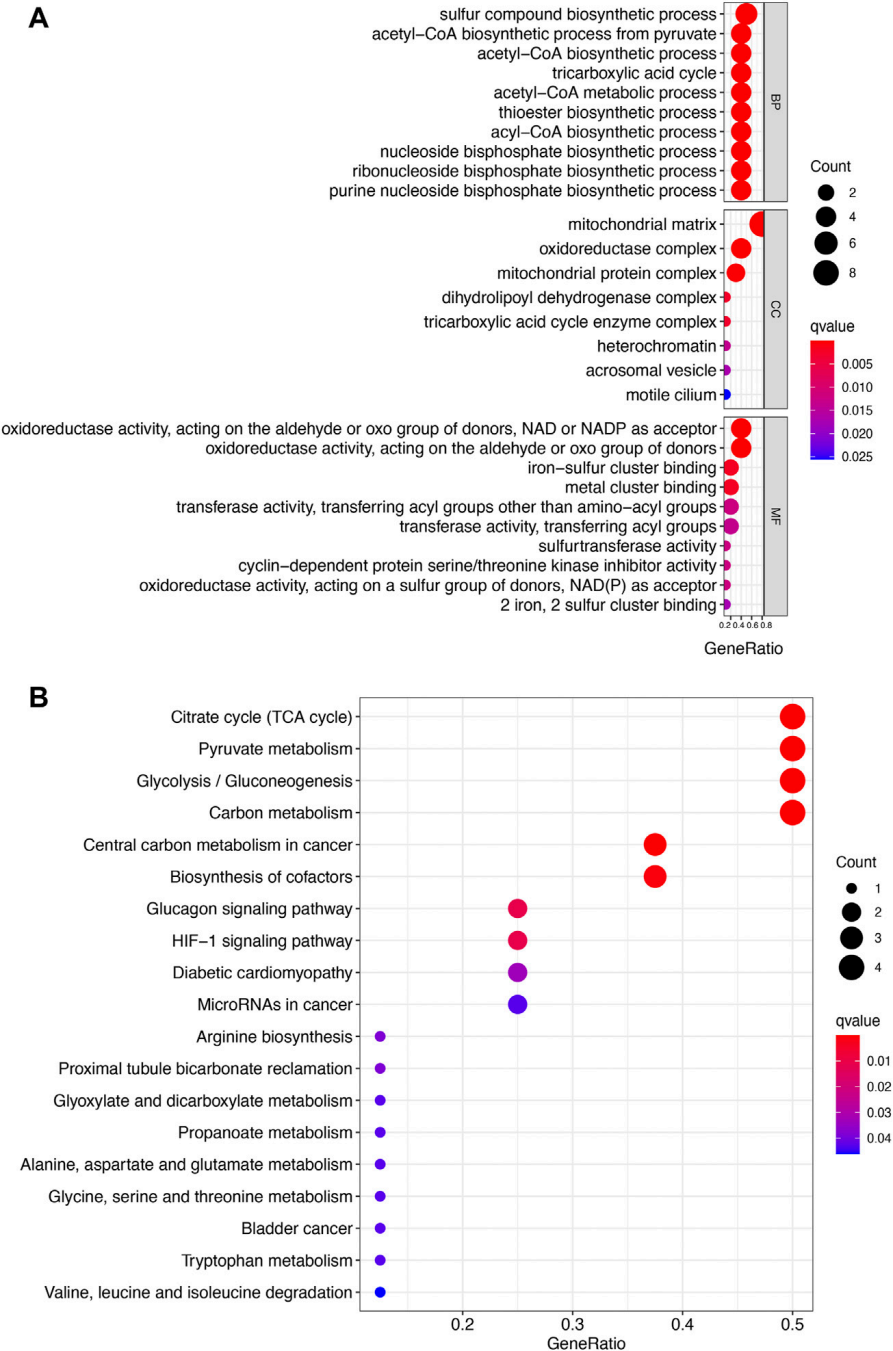
Enrichment analysis of cuproptosis-related genes in uterine corpus endometrial carcinoma. **(A)** Enriched items in gene ontology analysis. **(B)** Enriched items in Kyoto Encyclopedia of Genes and Genomes. BP, biological process; CC, cellular component; MF molecular function.

Figures 2A,B show the mutation landscape of CRGs in UCEC, revealing that 80 UCEC samples had a genetic mutation. Among these 10 CRGs, MTF1 was the gene with the highest frequency of mutation followed by PDHA1 and GLS (Figures 2A,B). In variant classification and SNV class, missense mutation and C > T ranked at the top, respectively (Figure 2B). CNV analysis suggested that more than half of 10 PRGs had homozygous amplification, whereas FDX1 and DLAT had a widespread homozygous deletion (Figure 2C). In functional enrichment analysis, the result of GO analysis was shown in Figure 3A, which reveals that these CRGs were enriched in sulfur compound biosynthetic process, acetyl-CoA biosynthetic and metabolic process, mitochondrial matrix, and metal cluster binding. Moreover, these CRGs were enriched in the TCA cycle, pyruvate metabolism, glycolysis, central carbon metabolism in cancer, HIF-1 signaling pathway, and miRNAs in cancer in the KEGG pathway analysis (Figure 3B).

### Development of a Cuproptosis-Related Prognostic Signature in Uterine Corpus Endometrial Carcinoma

**FIGURE 4:**
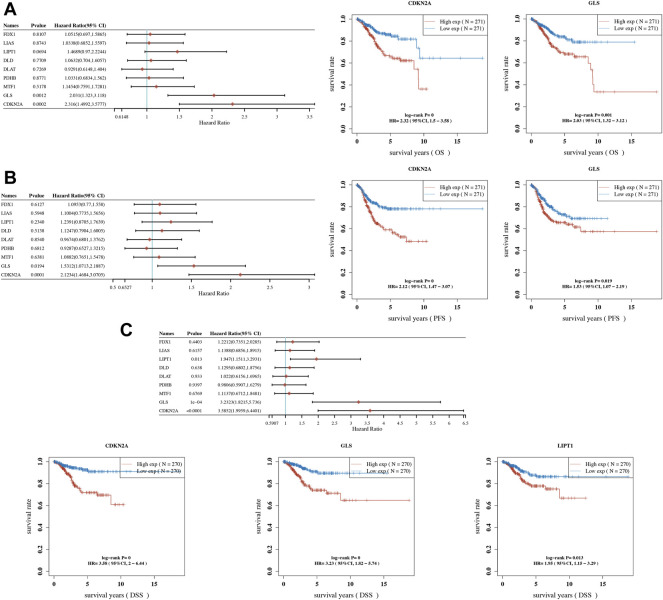
Prognostic significance of cuproptosis-related genes (CRGs) in uterine corpus endometrial carcinoma (UCEC). **(A)** Result of overall survival analysis of CRGs in UCEC. **(B)** Result of progression-free survival analysis of CRGs in UCEC. **(C)** Result of disease-free survival analysis of CRGs in UCEC. OS, overall survival; PFS, progression-free survival; DFS, disease-free survival.

**FIGURE 5:**
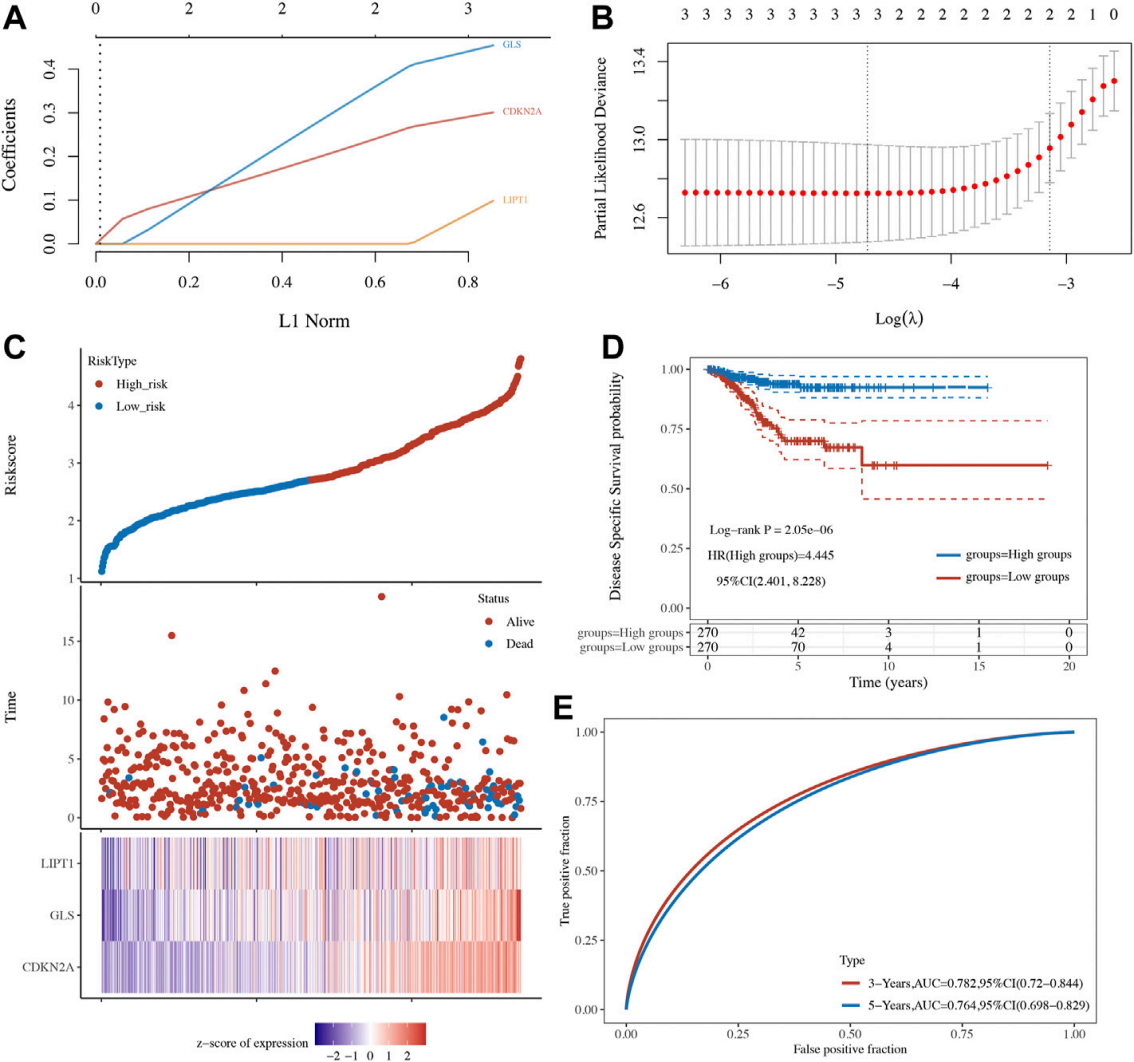
A cuproptosis-related prognostic signature in uterine corpus endometrial carcinoma (UCEC). **(A,B)** Coefficient and partial likelihood deviance of prognostic signature. **(C)** The risk score distribution, patient survival status, and cuproptosis-related gene expression profile of prognostic signature. **(D,E)** Survival curve of UCEC patients with high/low-risk score and ROC curve of prognostic signature.

**FIGURE 6:**
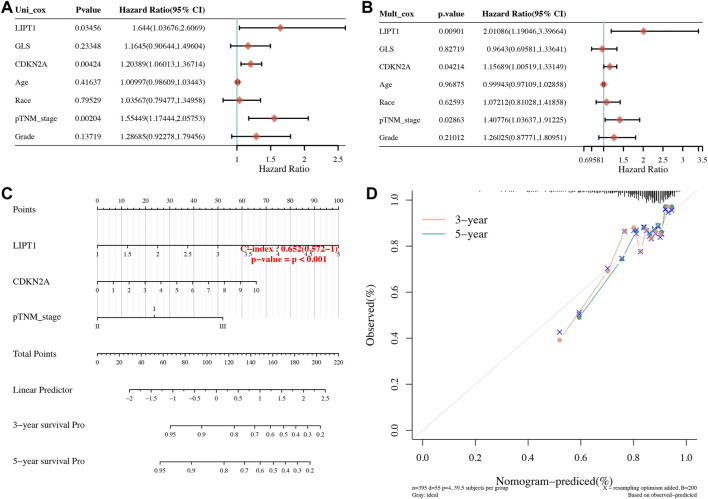
Development of a predictive nomogram. **(A,B)** Univariate and multivariate Cox regression considering clinical parameters and cuproptosis-related prognostic biomarkers. **(C,D)** Predictive nomogram to predict the 3- and 5-year survival of uterine corpus endometrial carcinoma patients. Calibration curve for the survival nomogram model in the discovery group. A dashed diagonal line represents the ideal nomogram.

OS, PFS, and DFS analyses were performed to identify a potential cuproptosis-related prognostic marker for UCEC. The result of OS analysis is shown in Figure 4A, which reveals that UCEC patients with high level of CDKN2A (*p* < 0.0001, HR = 2.32) and GLS (*p* = 0.001, HR = 2.03) had a poor OS rate. As shown in Figure 4B, PFS analysis suggested a poor OS rate in UCEC patients with a high level of CDKN2A (*p* < 0.0001, HR = 2.12) and GLS (*p* = 0.019, HR = 1.53). Furthermore, the result of PFS analysis demonstrated that high expression of CDKN2A (*p* < 0.0001, HR = 3.58), GLS (*p* < 0.0001, HR = 3.23), and LIPT1 (*p* = 0.013, HR = 1.95) had a poor prognosis in UCEC (Figure 4C). All in all, CDKN2A, GLS, and LIPT1 were potential prognostic biomarkers for UCEC. Based on these potential prognosis biomarkers, LASSO Cox regression analysis was performed and a cuproptosis-related prognostic signature including these three prognostic biomarkers (CDKN2A, GLS, and LIPT1) was constructed. Figures 5A,B reveal the coefficient and partial likelihood deviance of cuproptosis-related prognostic signature. After calculating the risk score of UCEC patients using the formula ((0.2725)*CDKN2A+(0.416)*GLS+(0.0143)*LIPT1), UCEC patients were divided into high- and low-risk groups and the risk score, survival status, and gene expression of prognostic signature was shown in Figure 5C. Moreover, the data revealed that UCEC patients with high risk scores had a poor prognosis (Figure 5D, *p* = 2.05e^−6^, HR = 4.45), with an area under the curve of 0.782 and 0.764 in a 3- and 5-year ROC curve (Figure 5E), demonstrating a good performance of this signature in predicting the prognosis of UCEC patients. Considering clinicopathologic characters and prognostic signature, univariate and multivariate analyses were performed to further identify the prognostic factors, which demonstrated CDKN2A, LIPT1, and pTNM stage as independent prognosis factors for UCEC patients (Figures 6A,B). A predictive nomogram was constructed based on these results, suggesting the good performance of this predictive nomogram in the 3‐ and 5‐year survival rates compared with an ideal model (Figures 6C,D).

### Risk Model Genes Analysis

**FIGURE 7:**
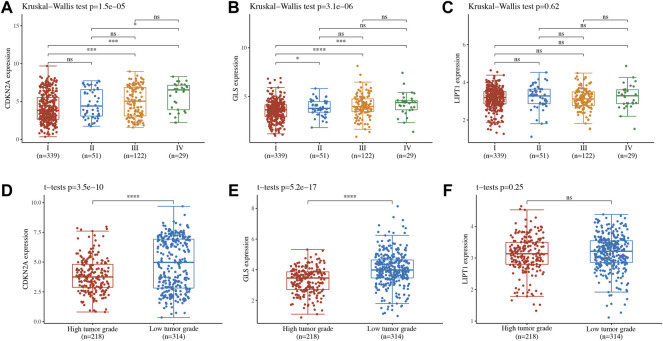
Expression of cuproptosis-related prognostic biomarkers in different groups of uterine corpus endometrial carcinoma (UCEC) patients. **(A–C)** Expression of CDKN2A, GLS, and LIPT1 in UCEC patients in different pTNM stages. **(D–F)** Expression of CDKN2A, GLS, and LIPT1 in UCEC patients in different tumor grades.

**FIGURE 8:**
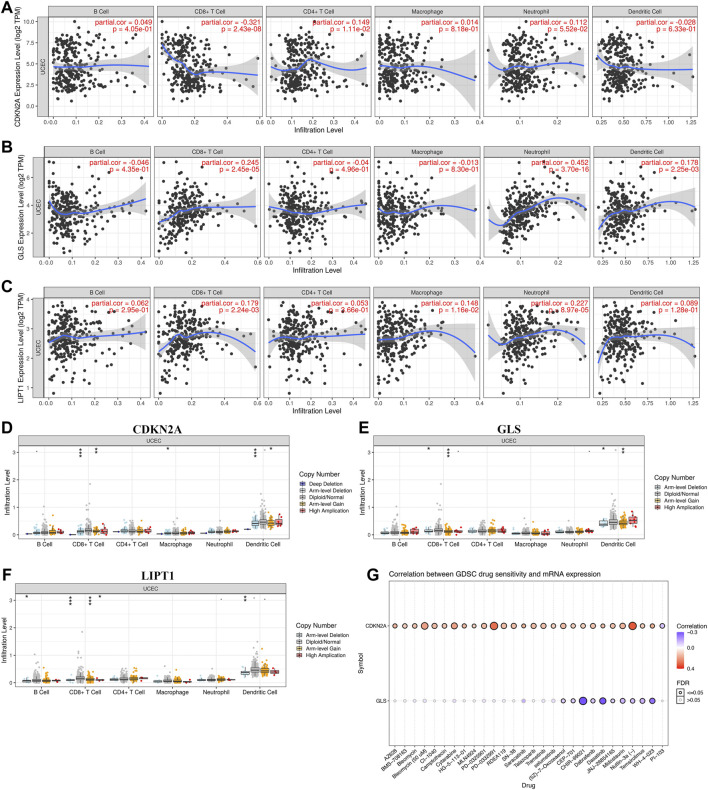
Immune cell infiltration and drug sensitivity analysis of cuproptosis-related prognostic biomarkers in uterine corpus endometrial carcinoma (UCEC). **(A–C)** Correlation between the expression of CDKN2A, GLS, and LIPT1 and immune cell infiltration in UCEC. **(D–F)** Correlation between copy number variation of CDKN2A, GLS, and LIPT1 and immune cell infiltration in UCEC. **(G)** Correlation between the expression of CDKN2A, GLS, and LIPT1 and drug sensitivity in UCEC. GDSC, genomics of drug sensitivity in cancer.

**FIGURE 9:**
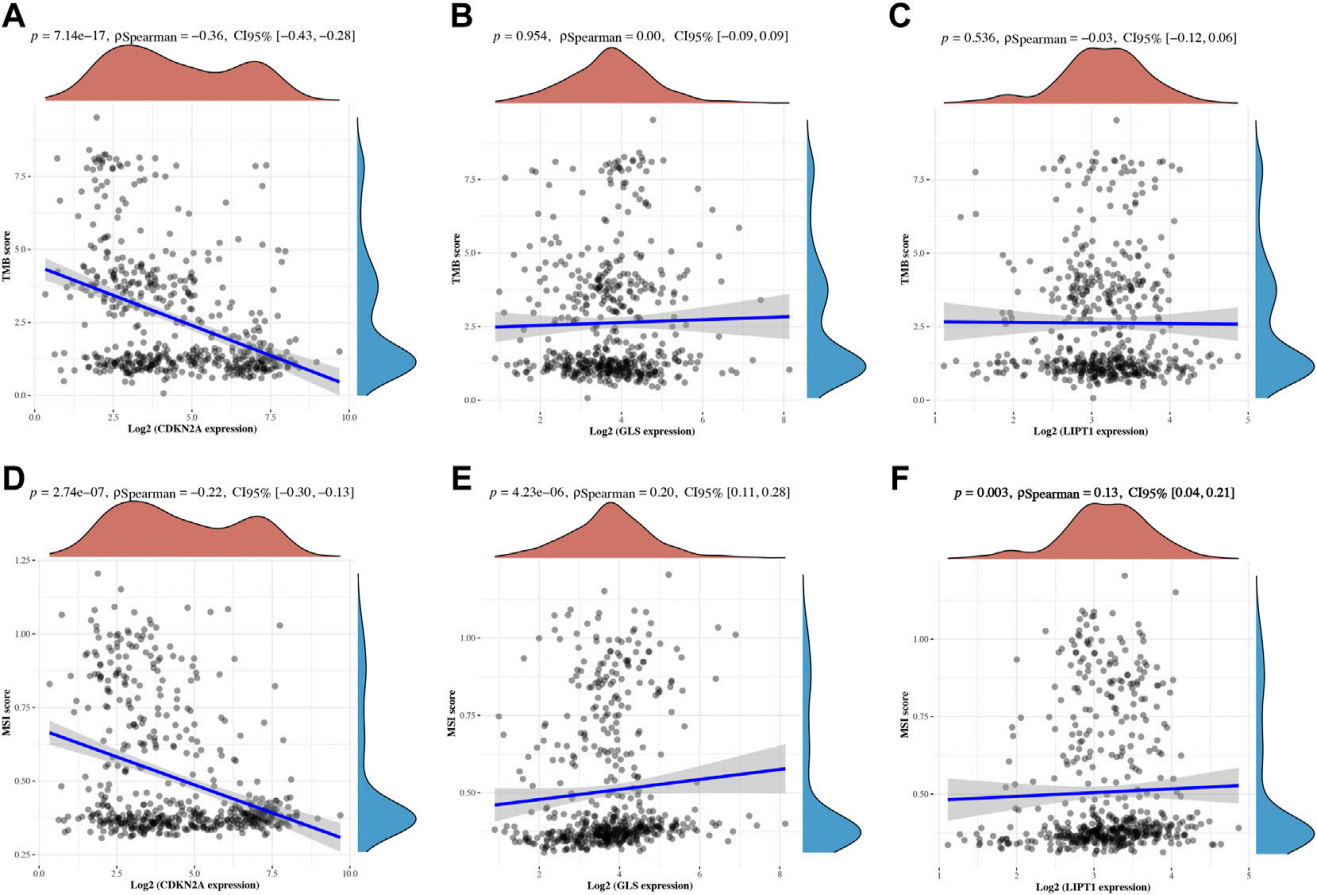
TMB and MSI of cuproptosis-related prognostic biomarkers in uterine corpus endometrial carcinoma (UCEC). **(A-C)** Correlation between the expression of CDKN2A, GLS, and LIPT1 and TMB score in UCEC. **(D–F)** Correlation between the expression of CDKN2A, GLS, and LIPT1 and MSI score in UCEC. TMB, tumor mutation burden; MSI, microsatellite instability.

The correlation between cuproptosis-related prognostic genes and tumor grade as well as pTNM stage was also analyzed. The expression of CDKN2A (Figure 7A, *p* = 1.5e^−5^), GLS (Figure 7B, *p* = 3.1e^−6^) increased as the pTNM stage increased in UCEC. Moreover, the expression of CDKN2A (Figure 7D, *p* = 3.5e^−10^) and GLS (Figure 7E, *p* = 5.2e^−17^) in UCEC patients with low tumor grade were higher than in those with high tumor grade. However, there is no significant correlation between LIPT1 expression and pTNM stage (Figure 7C, *p* = 0.62) and tumor grade (Figure 7F, *p* = 0.25). In immune infiltration analysis, CDKN2A expression increased as the abundance of CD8^+^ T cells decreased and the abundance of CD4^+^ T cells and neutrophils increased (Figure 8A). The result suggested a positive correlation between GLS expression and the abundance of CD8^+^ T cells, neutrophils, and dendritic cells (Figure 8B). Moreover, the result found that LIPT1 expression was positively correlated with the abundance of CD8^+^ T cells, macrophages, and neutrophils (Figure 8C). Somatic cell copy number alteration of CDKN2A, GLS, and LIPT1 could inhibit the infiltration level of some immune cells (Figures 8D–F). Drug sensitivity analysis was performed to explore the potential of CDKN2A, GLS, and LIPT1 as drug scanning targets for UCEC, showing that high CDKN2A expression and low GLS expression were positively correlated with drug resistance of GDSC (Figure 8G). Increasing pieces of evidence suggested TMB and MSI as predictive markers for tumor immunotherapy efficacy in cancer (Liu et al., 2019; Samstein et al., 2019). In the current study, the data indicated a negative correlation between TMB score and CDNK2A expression (Figure 9A, *p* = 7.14e^−17^) but not the expression of GLS (Figure 9B, *p* = 0.954) and LIPT1 (Figure 9C, *p* = 0.536). Moreover, the MSI score of UCEC patients increased as CDNK2A expression (Figure 9D, *p* = 2.74e^−7^) decreased and the expression of GLS (Figure 9E, *p* = 4.23e^−6^) and LIPT1 (Figure 9F, *p* = 0.003) increased. Above all, among these three genes, CDNK2A was the most possible gene involved in the progression of UCEC.

### Validation of the Expression of Risk Model Genes

As expected, the expression of CDKN2A (Supplementary Figure S2A) was upregulated, whereas the expression of GLS (Supplementary Figure S2B) and LIPT1 (Supplementary Figure S2C) was downregulated in UCEC cells versus normal human endometriosis cells. These were consistent with the above results.

### lncRNA–miRNA–mRNA ceRNA Network Analysis

**FIGURE 10:**
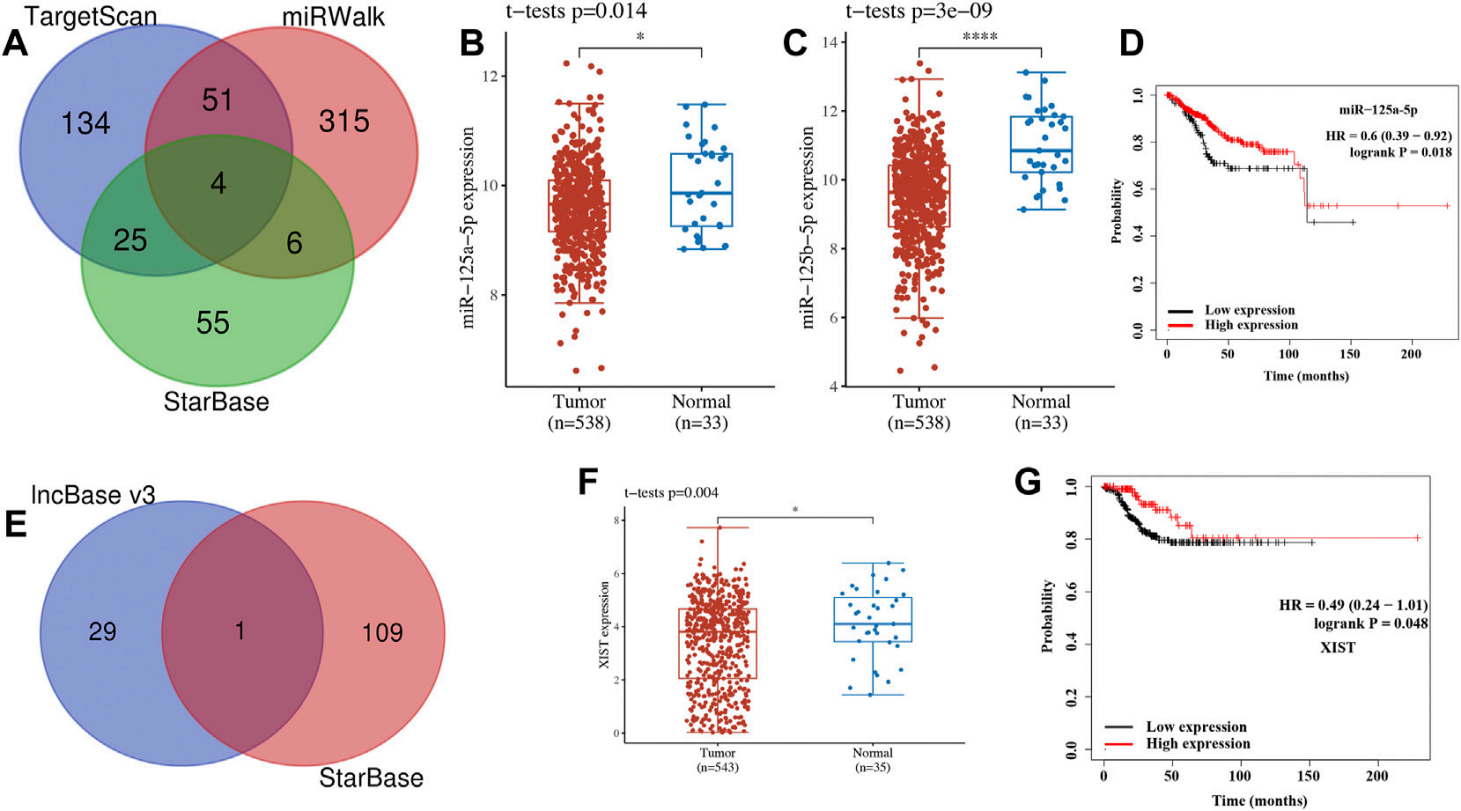
lncRNA–miRNA–mRNA regulatory axis in uterine corpus endometrial carcinoma (UCEC). **(A)** The miRNA targets predicted by TargetScan, miRWalk, and StarBase. **(B–D)** The expression and prognosis significance of miRNA target in UCEC. **(F)** The lncRNA targets predicted by lncBase and StarBase. **(F,G)** Expression and prognosis significance of lncRNA XIST in UCEC.

To further clarify the hub gene-associated mechanism in UCEC, a lncRNA–miRNA–mRNA ceRNA network analysis was performed. Combined with the predicted result of StarBase, miRWalk, and TargetScan, a total of four miRNAs (miR-125a-5p, miR-125b-5p, miR-4319, and miR-485-5p) were identified as the most potential miRNA targets of CDNK2A (Figure 10A). However, only two of four miRNAs were differently expressed in UCEC (Figures 10B,C). To be more specific, the expression of miR-125a-5p (Figure 10B, *p* = 0.014) and miR-125b-5p (Figure 10C, p = 3e^−9^) were downregulated in UCEC versus normal tissues. Further OS revealed that UCEC patients with high miR-125a-5p levels had better survival (Figure 10D, *p* = 0.018). Thus, miR-125a-5p was the most possible miRNA target of CDKN2A. The lncRNA target of miR-125a-5p was further explored. Combined with the predicted result of lncBase V3 and StarBase, lncRNA XIST was suggested as the most potential target of miR-125a-5p (Figure 10E). Further analysis revealed that XIST expression was downregulated in UCEC (Figure 10F, *p* = 0.004) and high XIST levels had a better survival (Figure 10G, *p* = 0.048). Thus, lncRNA XIST/miR-125a-5p/CDKN2A regulatory axis may play a vital role in the progression of UCEC and further *in vivo* and *in vitro* studies should be conducted to verify this hypothesis.

## Discussion

Characterized by biological invasiveness and potent metastasis, UCEC is still a major problem affecting public women’s health (Li et al., 2021). Moreover, the disadvantage of it was that the incidence and mortality of UCEC are increasing (Amant et al., 2005). There are few satisfactory biomarkers or approaches that could predict the prognosis of UCEC patients accurately. Since the significant role exerted by cuproptosis in the oncogenesis, severity, and development of malignancy, it is important to explore the significance of CRGs in the development and prognosis of UCEC.

Gene expression profile suggested that the expression of FDX1, LIAS, DLAT, and CDKN2A were upregulated, whereas the expression of LIPT1, DLD, PDHB, MTF1, and GLS were downregulated in UCEC versus normal tissues. These data were consistent with the results of previous studies. A previous study revealed that CDKN2A was upregulated in UCEC and it might be implicated in the pathogenesis of UCEC (Su et al., 2015). However, no study had been conducted to elucidate the expression and function of the other CRGs in UCEC. Correlation analysis demonstrated that the majority of CRGs were positively correlated with each other. GLS and PDHA1 played a synergistic role in promoting greater glutamine dependence in prostate cancer patients (Li et al., 2016). Moreover, PDHA1, PDHB, DLAT, and DLD played a synergistic role in pyruvate dehydrogenase complex deficiency (Inui et al., 2022). GO and KEGG analyses revealed that these CRGs were enriched in the TCA cycle, glycolysis, and HIF-1 signaling pathway. These pathways have been found to be involved in the progression and therapy of UCEC. By regulating the HIF-1 signaling pathway, AGR2-induced glucose metabolism could promote the progression of UCEC (Gong et al., 2020). HIF-1 signaling pathway-related hypoxia and hyperglycemia were associated with metformin resistance in endometrial cancer (Sivalingam et al., 2020). TCA cycle and its enzyme components play a vital role in basal cell metabolism, thus affecting tumor cell proliferation and invasion in UCEC (Iplik et al., 2018).

Further prognosis revealed that UCEC patients with high levels of CDKN2A and GLS had a poor OS rate. PFS analysis suggested a poor OS rate in UCEC patients with a high level of CDKN2A and GLS. Moreover, the result of the PFS analysis demonstrated that high expression of CDKN2A, GLS, and LIPT1 had a poor prognosis in UCEC. All in all, CDKN2A, GLS, and LIPT1 were potential prognostic biomarkers for UCEC. Another study also suggested CDKN2A as a prognostic biomarker in human endometrial cancer (Zhang et al., 2020). Chen et al. (2021b) found that LIPT1 was associated with survival in urothelial cancer. Moreover, GLS differentially could modulate the prognosis of human cancer (Saha et al., 2019). Furthermore, LASSO Cox regression analysis was performed and identified a cuproptosis-related prognostic signature including these three prognostic biomarkers (CDKN2A, GLS, and LIPT1), which had a good performance in predicting the prognosis of UCEC patients. As far as we know, the TNM system was the most reliable predictive method for the prognosis of UCEC patients. Although some prognostic signatures had been identified for the prognosis of UCEC patients (Hu et al., 2021; Weijiao et al., 2021), these prognostic signatures had not been used for the prognosis of UCEC patients clinically. The current study identified the first cuproptosis-related prognostic signature for UCEC patients, which may be used for the prognosis prediction of UCEC patients clinically.

In immune infiltration analysis, the result revealed that the expression of CDKN2A, GLS, and LIPT1 was positively correlated with the abundance of certain immune cells, including CD8^+^ T cells and neutrophils. Similar results had been reported in other types of cancers. CDKN2A expression was positively correlated with infiltrating levels into CD8^+^ T cells, CD4^+^ T cells, and neutrophils in hepatocellular carcinoma (Luo et al., 2021). Another integrative bioinformatics analysis revealed that CDKN2A shows a significant correlation with immune signatures in multiple myeloma, including CD4^+^ regulatory T cells, T cell exhaustion, and neutrophils (Tuerxun et al., 2022). A previous study revealed that STAT5 and CDKN2A/CDKN2B could promote the proliferation and terminal differentiation of CD8^+^ T cells (Grange et al., 2015).

Another important finding of the current study was that we identified that lncRNA XIST/miR-125a-5p/CDKN2A regulatory axis may be involved in the progression of UCEC. Increasing pieces of evidence had revealed that lncRNA XIST played a vital role in many biological processes in human cancer (Liu et al., 2018; Zhu et al., 2018; Yang et al., 2020b). Overexpression of miR-125a-5p could inhibit tumor cell apoptosis in UCEC (Shi et al., 2019). CDKN2A was involved in tumor cell growth and drug resistance in human breast cancer (Lubecka et al., 2018). A previous study revealed that miR-125a-5p/CDKN2A regulatory axis exerts a vital role in prognostic value in cervical cancer (Wang et al., 2021). The development and progression of UCEC was a complex process with continuous progression and multiple steps. The specific molecular mechanism of UCEC had not been fully elucidated. In the current study, a lncRNA XIST/miR-125a-5p/CDKN2A regulatory axis was identified for UCEC and it has not been studied before, which provided another theoretical basis for the further study of the mechanism of oncogenesis and progression of UCEC.

The current study also had some limitations. Cuproptosis-related prognostic signature should be verified by another dataset. Further *in vivo* and *in vitro* studies should be conducted to verify the lncRNA XIST/miR-125a-5p/CDKN2A regulatory axis.

## Conclusion

In conclusion, the current study identified a cuproptosis-related prognostic signature including these three prognostic biomarkers (CDKN2A, GLS, and LIPT1) for UCEC. The ceRNA network also identified that lncRNA XIST/miR-125a-5p/CDKN2A regulatory axis may be involved in the progression of UCEC. Further *in vivo* and *in vitro* studies should be conducted to verify these results.

## Data Availability

The original contributions presented in the study are included in the article/Supplementary Material, further inquiries can be directed to the corresponding author.
