# Supplementary figures and images for: Identification and Validation of Cuproptosis-Related Prognostic Signature and Associated Regulatory Axis in Uterine Corpus Endometrial Carcinoma
Source: Front Genet. 2022 Jul 22;13:912037. doi: 10.3389/fgene.2022.912037 (PMC9353190; doi:10.3389/fgene.2022.912037)

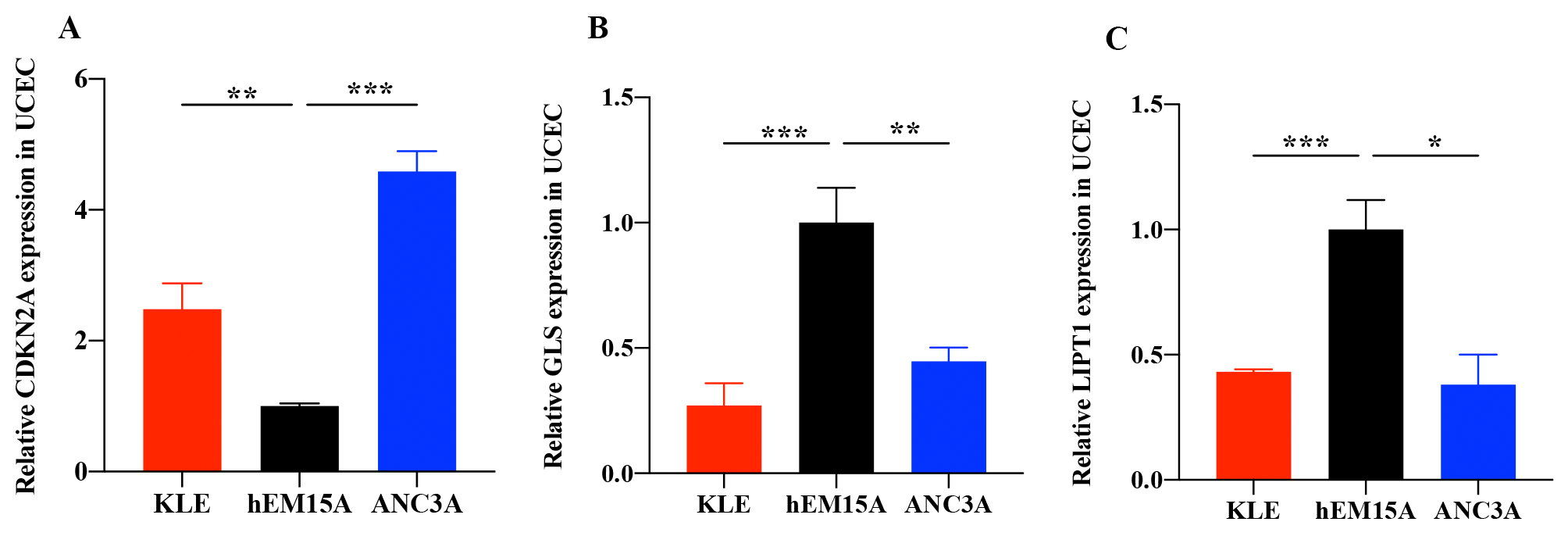

Supplement: Supplementary file 1 [file Image2.TIF]

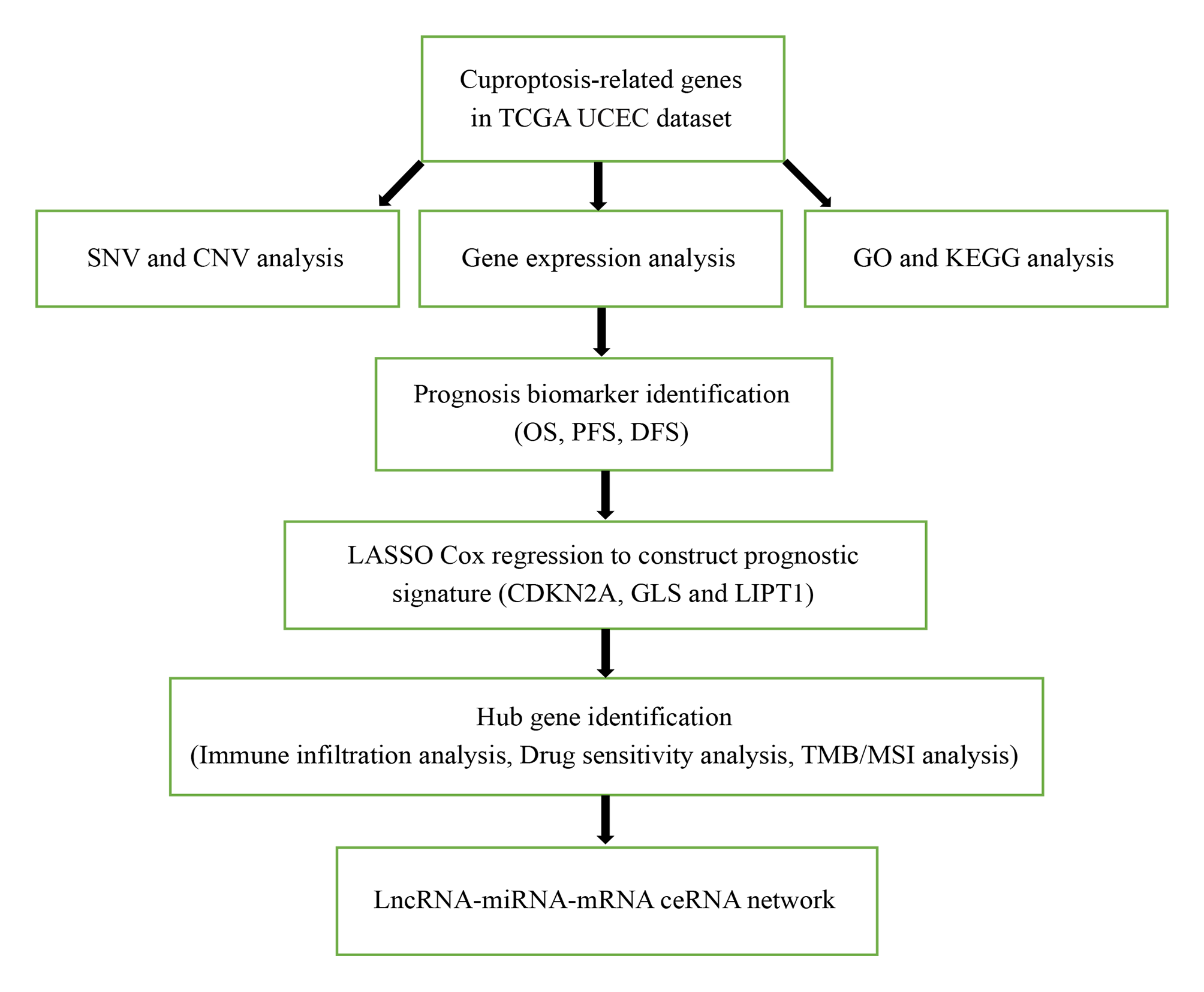

Supplement: Supplementary file 2 [file Image1.TIF]
